# The need to address the impact of COVID-19 on TB control for vulnerable groups

**DOI:** 10.5588/pha.22.0022

**Published:** 2022-09-21

**Authors:** Z. Aranda, H. J. Sánchez-Pérez

**Affiliations:** 1 Partners In Health Mexico/Compañeros En Salud, Ángel Albino Corzo, México; 2 Departamento de Salud, El Colegio de la Frontera Sur, San Cristóbal de las Casas, México; 3 Grup de Recerca d’Amèrica i Àfrica Llatines, Cerdanyola del Vallés, Catalunya, Spain

Dear Editor,

We would like to call on health providers, researchers and decision-makers to develop studies and interventions that address the impact of COVID-19 on TB control in vulnerable populations. This is inspired by the recent article by Dheda and colleagues in the *Lancet Respiratory Medicine*,^1^ in which the authors describe the effects of the pandemic on TB control at the global and national levels, but without acknowledging the heterogeneous impact among populations with different sociodemographic characteristics. Alarmed by this omission, we wanted to highlight the need to address the particularly marked impact of COVID-19 on TB care for vulnerable groups. This is exemplified by the current situation affecting indigenous and migrant populations living in the state of Chiapas, Mexico.

Chiapas is the southernmost state of Mexico, neighbouring Guatemala. The state has the second highest proportion of speakers of indigenous languages in the country (28.2%),^2^ and serves as an entry point into Mexico for thousands of migrants each year, mainly from Central America and the Caribbean, with 70% of all national refugee applications concentrated in a single municipality.^3^ The migrant population in Chiapas has almost doubled over the past 10 years due to political instability, social violence, natural disasters and economic hardship – aggravated by the COVID-19 pandemic – in their countries of origin. In 2018, indigenous populations among the citizens of Chiapas had the highest levels of poverty (above 90%) and lack of access to health services (above 20%),^2^ exacerbated by widespread racism and a lack of cultural competence among health providers. In the case of migrant populations, almost half had left their home countries due to economic issues and less than 50% had received healthcare when needed.^4^ It is well known that poorer living conditions, hygiene and health support among indigenous and migrant citizens lead to increased susceptibility to TB, and poorer diagnosis, management and prognosis of the disease in these populations. On top of this, the COVID-19 pandemic has increased economic hardship and limited access to health services for vulnerable groups in Chiapas, who have been disproportionately affected by the COVID-19 disease burden, thereby worsening the state of TB and TB care among indigenous peoples and migrants. For example, TB case detection in indigenous populations declined by 50% from 2019 to 2020 in one of 15 regions in Chiapas, compared to a 30% decrease in the general population.^5^

This example highlights the importance of gathering evidence on the specific effect of the COVID-19 pandemic on TB control in vulnerable groups. This is often overshadowed by national-level data and demonstrates the need to develop intersectoral interventions to tackle the social determinants that increase susceptibility to TB and impede access to TB care among populations that are particularly affected by the pandemic.
